# Long-Term Hand and Shoulder Function in Children following Early Surgical Intervention for a Birth-Related Upper Brachial Plexus Injury

**DOI:** 10.1055/s-0044-1787151

**Published:** 2024-06-21

**Authors:** Rachel N. Aber, Leslie A. Grossman, Aaron J. Berger, Andrew E. Price, Israel Alfonso, John A.I. Grossman

**Affiliations:** 1 Sackler School of Medicine, Tel Aviv University, Tel Aviv, Israel; 2 Brachial Plexus Program, Nicklaus Children's Hospital, Miami, Florida, United States; 3 Nicklaus Children's Hospital, Miami, Florida, United States; 4 Orthopedic Surgery, NYU Langone Medical Center, New York, New York, United States

**Keywords:** brachial plexus birth injury, hand function, long-term outcomes, surgery

## Abstract

**Purpose** To better understand the long-term hand and shoulder outcomes of upper brachial plexus birth injuries.

**Methods** We evaluated shoulder and hand function in 32 patients (13 males; 19 females) with a C5/C6 birth injury history). All patients had undergone primary nerve surgery as infants, and 12 underwent a simultaneous shoulder procedure as they presented with a fixed internal rotation contracture of the shoulder. On average, all patients were evaluated and examined 15 years postoperatively. The shoulder function was evaluated using the Miami Shoulder Scale. Hand function was measured by the 9-hole peg test (9-HPT) and statistical analysis included comparison of 9-HPT time against normative data using the Student's *t* -test.

**Results** The cohort includes 22 right-hand-dominant and 10 left-hand-dominant patients. Mean age at surgery was 10 months; mean age at follow-up was 15 years ± 2 years 2 months. Cumulative shoulder function was “good” or “excellent” (Miami score) in 23 patients. For 9-HPT, 23 out of 32 patients seen had an involved hand with a significant alteration in function.

**Conclusion** Early nerve surgery in cases of upper brachial plexus birth injuries result in the desired outcome. To ensure timely and targeted therapy for any residual deficits, it is imperative that limitations in hand function among children with an Erb's palsy.

## Introduction

Most birth-related injuries to the brachial plexus involve spinal nerves C5 and C6, which merge into the upper trunk of the plexus and are commonly known as Erb's palsy. 1 2 The upper trunk is responsible for the innervation of the shoulder muscles. Thus, management and evaluation of the shoulder are often of primary concern in these patients. The shoulder deficit is a marker of the degree of injury. 3 4 Spontaneous recovery with minimal impairment is seen in most patients with a C5–C6 lesion, ranging from approximately 50% to over 95% of patients. 5 6 The remaining patients (10–30%) suffer permanent impairment of shoulder function, regardless of treatment. 7 8 9 10 Surgical intervention is generally accepted for patients with an upper/middle (C5/C6/C7) injury and global palsies 5 ; however, there has been more uncertainty surrounding early nerve reconstruction for upper brachial plexus birth injuries (BPBI).

More recently, attention has been drawn to limitations of hand function in patients with upper BPBI, contrary to the accepted dogma that the hand remains unaffected. 2

In this study, we report long-term outcomes in children with C5–C6 pattern injuries who underwent early nerve reconstruction.

## Methods

**Fig. 1:**
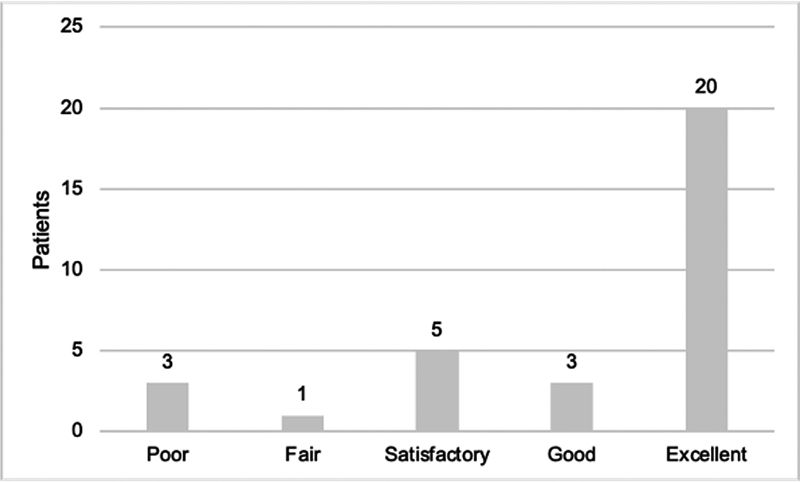
Miami classification. Shoulder evaluation: C5, C6 repair long-term follow-up ( *n* = 32).

A retrospective chart review of patients undergoing primary nerve reconstruction performed by the senior author for Erb's palsy between 2000 and 2005 was conducted. Of the 131 patients who received early surgical intervention, 55 were identified as having an upper BPBI. From this cohort of 55 patients with upper BPBI requiring reconstruction, 32 (58.2%) were available for long-term follow-up. These patients underwent clinical evaluation, including calculation of shoulder function by Mallet grading system and Miami shoulder score as documented in Table 1 of this article. For reference we have also included the Miami shoulder score classification system in Fig. 1 . A 9-hole peg test (9-HPT) was performed on each patient, which was used to determine hand function, and which had been previously validated in 2012 2 ( Table 2 ).

**Table 1:** Miami shoulder classification: total grade for active abduction/forward flexion and external rotation

Average active abduction/forward flexion (degrees)	Active external rotation (degrees)	Total score a	Grade
0	Fixed deformity	0	0 (no function)
<45	Full passive <10	1–2	1 (poor)
<90	<30	3–4	2 (fair)
90–120	30–45	5–6	3 (satisfactory)
120–150	45–90	7–8	4 (good)
>150	90	9–10	5 (excellent)

a Maximum shoulder score =10; decrease score by 1 point for a contracture of >20 degrees.

**Table 2:** Aggregate results of the nine-hole peg test

	9-HPT involved hand(s) (mean of attempts)	9-HPT uninvolved hand(s) (mean of attempts)	Difference in performance(s) (involved − uninvolved) a	Percent difference (percent of uninvolved) b	Normative mean(s) (involved hand) b	Normative mean(s) (uninvolved hand) b	Normative Differences involved − uninvolved)	Normative percent difference (percent of uninvolved) a
**Mean**	25.2	22.4	2.9	12.8	17.7	17.0	0.68	4.2
**SD**	5.5	4.4	3.1	13.6	0.87	0.96	0.96	5.66
**Range**	19 to 48	18–42	−4 to 11	−14 to 43	17–19	16–18	−1 to 2	−7 to 14

Abbreviations: 9-HPT, 9-hole peg test; SD, standard deviation.

a Percent data were rounded to the nearest total percentage.

b Normative data were extracted from Poole et al and are sex, age, and hand dominance matched. Discrepancies between the involved and the uninvolved extremities are recorded as positive when the involved hand was worse (longer time to complete the test) and negative when it was better (shorter time to complete the test).

Basic analysis, including mean and standard deviation (SD), was performed on multiple measures, as reported below ( Table 2 ). Comparisons were made to the available normative population mean times matched for age, sex, and hand dominance. Statistical analysis was performed using the Student's *t* -test.

## Results

Of the 55 patients with C5–C6 injuries that underwent primary nerve reconstruction, 32 patients were available for long-term follow-up. We detail the demographic makeup and surgical history of all patients seen in follow-up. There were 13 males and 19 females. The mean age at surgery was 10 months (SD = 4.5 months). We do not generally operate in our program on pure C5/C6/upper trunk lesions until 8 months of age except in unusual circumstances. The mean age at the final follow-up was 15 years (SD = 2 years 2 months; Supplementary Tables S1 and S2 , available in online version only). Supplementary Tables S3 and S4 (available in online version only) include the information available to the authors about the patients not seen in long-term follow-up.

All patients underwent primary nerve surgery. The patients who presented with a shoulder contracture or loss of full passive external rotation of the shoulder when examined under anesthesia had a simultaneous surgical correction. Seven patients reported that the involved extremity was their dominant hand (two left-handed and five right-handed patients). The remainder reported that the involved hand was nondominant. In Table 1 , cumulative shoulder function was evaluated by Miami score as “good” or “excellent” in 23 individuals and 4 individuals received a “poor” analysis of shoulder function. The mean Miami score was 4.1 (SD = 1.3; Fig. 1 ).

9-HPT performance times are reported in Table 2 . Twenty-three of the 32 patients took longer to complete the task using the involved hand than the uninvolved hand. Eighteen of these patients are right-hand dominant, and five are left-hand dominant. The mean time difference between the involved and the uninvolved extremities was 2.9 seconds, compared with the expected difference of 0.68 seconds (based on age-, sex-, and hand dominance-matched normative data). 11 This difference was statistically significant ( *p* = 0.00035). On average, individuals took 12.8% longer to complete the test with the involved hand; this was significantly different from the expected difference of 4.2% ( *p* = 0.001).

Two of the seven patients with hand dominance on the affected side demonstrated a markedly poorer score for the involved hand. The mean time difference for these five individuals was 2.7 seconds (SD = 5.3), compared with 2.9 seconds (SD = 2.3) for the remainder of the cohort (not significant). Interestingly, three patients in this cohort displayed no time difference between involved and uninvolved hands by 9-HPT.

## Discussion

We report on both long-term shoulder range of motion and fine motor hand function in children treated for upper brachial plexus birth palsy. We used the 9-HPT as a validated outcome tool for the hand. The majority of the individuals in our cohort had detectable hand function deficits. The persistent hand deficits seen in our study are contrary to the accepted “normal” hand function in individuals with a C5–C6 lesion. In 2012, our group published similar findings with a much smaller cohort with a shorter follow-up time. 2

In our current study, the majority of children reported their noninjured hand to be dominant. If the function of the two hands is equal, hand dominance is likely determined by a mechanism unrelated to the brachial plexus injury. The reported prevalence of left-handedness in the general population is 8 to 10%; in our cohort, it is 31.2%. This suggests that hand dysfunction can affect hand dominance, as it is likely that some of the children selectively became left-handed to compensate for a persistent functional deficit in their right hand.

One possible limitation of this study is that the 9-HPT requires active shoulder function to position the hand to execute the test. Residual shoulder deficit might affect the results. However, most individuals had “excellent” shoulder function on the Miami shoulder scale ( Table 1 ). No correlation was observed between the shoulder scores and the 9-HPT score.

Our data were derived from two attempts by each child individually. Each normative data point was obtained by testing 21 to 43 children, with each child first performing a practice followed by a timed test. 11 Therefore, the normative data are an average of many children performing the test. These findings define the expected “norm” for the population of a given sex, age, and hand dominance. We intended to compare our participants involved and noninvolved hands to what would be expected in an unaffected age-matched population. However, the normative data did not provide specific hand-to-hand comparisons for individual children, only an aggregate for the tested group's left- and right-hand times. It seems unlikely that typically developing children would exhibit striking differences in performance between the dominant and nondominant hands, so we believe that our comparison is still valid.

## Conclusion

Most patients in our cohort demonstrated long-term deficits in hand function with good or excellent shoulder function, challenging the generally accepted notion that C5–C6 pattern birth injuries do not influence hand function. Injuries that involve the upper brachial plexus exclusively may include subtle unrecognized injuries to the lower brachial plexus as well. Early referral and implementation of multidisciplinary strategies allow these children the best chance of functional recovery from upper brachial plexus injury. Further study of hand function is warranted in these individuals to redirect early treatment strategies.
