# Supplementary material for: Long-Term Hand and Shoulder Function in Children following Early Surgical Intervention for a Birth-Related Upper Brachial Plexus Injury
Source: J Brachial Plex Peripher Nerve Inj. 2024 Jun 21;19(1):e27–30. doi: 10.1055/s-0044-1787151 (PMC11192585; doi:10.1055/s-0044-1787151)
Supplement: Supplementary file 1 — Supplementary Material [file 10-1055-s-0044-1787151-s2300006.pdf]

Supplementary Table S1 Demographic summary of patients seen in long-term follow-up[6]

	Mean birth weight	Mean age of surgery (mo)	Mean age at follow-up (y)	Hand dominance and injured side
Males ($n=13$)	9 lbs and 0 oz	9	15	RD: 10 ; 6 LBPI ; 4 RBPI<br>LD: 3 ; 3 RBPI
Females ($n=19$)	8 lbs and 6 oz	11	15	R: 13 ; 12 LBPI ; 1 RBPI<br>L: 6 ; 2 LBPI ; 4 RBPI
Total ($n=32$)	8 lbs and 7 oz	10	15	R: 22 ; 18 LBPI ; 5 RBPI<br>L: 10 ; 2 LBPI ; 7 RBPI

Abbreviations: LBPI, left-sided brachial plexus injury; LD, left hand dominance; RBPI, right-sided brachial plexus injury; RD, right hand dominance.

Supplementary Table S2 Type of surgical nerve repair performed on patients seen in long-term follow-up

Type of nerve repair[a]	No. patients ($n=32$)
Neurolysis	32
Cervical plexus graft	7
Sural graft	23
Spinal accessory transfer	2
Simultaneous primary shoulder repair	12
Simultaneous Botox injection	27
Secondary shoulder procedure	13
Secondary Botox injection (s)	15

[a]All patients received a primary neurolysis. Additional primary procedures were determined based on clinical examination and preoperative and intraoperative findings. Secondary repairs were determined on the severity of the injury, postoperative function, and clinical examination.

Supplementary Table S3 Demographics summary of patients not seen in long-term follow-up[a]

	Mean birth weight	Mean age of surgery (mo)
Males ($n=13$)	8 lbs and 9 oz	12
Females ($n=10$)	9 lbs and 4 oz	13
Total ($n=23$)	9 lbs and 2 oz	12

[a]Demographic information available on this subset of patients at the time of the study.

Supplementary Table S4 Type of surgical nerve repair performed on patients not seen in long-term follow-up

Type of nerve repair[a]	No. patients ($n=23$)
Neurolysis	23
Cervical plexus graft	9
Sural graft	10
Spinal accessory transfer	4
Simultaneous primary shoulder repair	8
Simultaneous Botox injection	21
Secondary shoulder procedure	4
Secondary Botox injection (s)	6

[a]All patients received a primary neurolysis. Additional primary procedures were determined based on clinical examination and preoperative and intraoperative findings. Secondary repairs were determined on severity of the injury, postoperative function, clinical examination, and during limited follow-up.

 © 2024. The Author(s).
